# Variation in human cancer cell external phosphatidylserine is regulated by flippase activity and intracellular calcium

**DOI:** 10.18632/oncotarget.6045

**Published:** 2015-10-09

**Authors:** Subrahmanya D. Vallabhapurapu, Víctor M. Blanco, Mahaboob K. Sulaiman, Swarajya Lakshmi Vallabhapurapu, Zhengtao Chu, Robert S. Franco, Xiaoyang Qi

**Affiliations:** ^1^ Division of Hematology and Oncology, Department of Internal Medicine, University of Cincinnati College of Medicine, Cincinnati, Ohio, USA; ^2^ Divison of Human Genetics, University of Cincinnati College of Medicine, Cincinnati, Ohio, USA

**Keywords:** phosphatidylserine, surface exposure, cancer cell biomarker, flippase, calcium

## Abstract

Viable cancer cells expose elevated levels of phosphatidylserine (PS) on the exoplasmic face of the plasma membrane. However, the mechanisms leading to elevated PS exposure in viable cancer cells have not been defined. We previously showed that externalized PS may be used to monitor, target and kill tumor cells. In addition, PS on tumor cells is recognized by macrophages and has implications in antitumor immunity. Therefore, it is important to understand the molecular details of PS exposure on cancer cells in order to improve therapeutic targeting. Here we explored the mechanisms regulating the surface PS exposure in human cancer cells and found that differential flippase activity and intracellular calcium are the major regulators of surface PS exposure in viable human cancer cells. In general, cancer cell lines with high surface PS exhibited low flippase activity and high intracellular calcium, whereas cancer cells with low surface PS exhibited high flippase activity and low intracellular calcium. High surface PS cancer cells also had higher total cellular PS than low surface PS cells. Together, our results indicate that the amount of external PS in cancer cells is regulated by calcium dependent flippase activity and may also be influenced by total cellular PS.

## INTRODUCTION

Although extensive research in the last few decades has led to greater understanding of cancer cell biology and complexity [1-5], cancer is still one of the major causes of death worldwide. Critical changes have been identified in key growth factor receptors, kinases, molecules associated with cell death regulation, adaptor proteins involved in cell signaling, and metabolic pathways [1-5], but effective cancer therapies have not yet materialized. Current cancer treatment with surgery, radiation and chemotherapy often affects normal cells and tissues while giving little survival benefit due to the recurrence of tumor after treatment [6-10]. Therefore, identification of novel therapeutic biomarkers to specifically target cancer cells while minimizing detrimental effects to normal cells is critical.

Compared to other areas of cancer cell biology, not much is known about the alterations in membrane lipids in cancer cells and how lipids are exploited by cancer cells for their growth and maintenance. In light of this, identification of elevated PS exposure on the membrane surface of viable, non-apoptotic cancer cells is of extreme biological and therapeutic interest and understanding the mechanism(s) involved in surface PS exposure will enhance our ability to effectively target and treat cancer [11-15]. Recently, our lab has shown that SapC-DOPS nanovesicles composed of saposin C (SapC) and dioleoylphosphatidylserine (DOPS), which recognize PS, specifically target and kill tumor cells both *in vitro* and *in vivo* [15-23]. In xenograft mouse models of cancer, the anti-tumor activity of SapC-DOPS occurred without toxic effects on normal cells or organs [15, 18, 19, 23]. SapC has natural affinity for PS at acidic pH [24-28] and hence selectively targets surface PS in the acidic microenvironment of tumors [15, 18, 19, 22, 23, 26-28].

In the plasma membrane of normal healthy cells, lipids are asymmetrically distributed across the inner and outer leaflets, with PS located predominantly on the inner leaflet [29, 30]. PS on the inner leaflet of the plasma membrane has essential roles in the activation of key kinases like PKC, PDK1, and Akt and serves as an interacting molecule for various signaling proteins [29, 31]. However, during certain physiological conditions like induction of cell death by apoptosis, activation of platelets to initiate blood clotting, activation of mast cells, etc., the asymmetrical distribution of PS is disturbed and PS is transported to the outer leaflet of the plasma membrane where it serves essential functions [32-35]. For instance, on apoptotic cells, exposed PS serves as a signal for macrophages to engulf dying cells [34-36]. Under normal physiological conditions, the asymmetrical distribution of PS is regulated by flippases (also known as aminophospholipid translocases) [37-43]. Flippases are inhibited by calcium and translocate PS from the outer to the inner leaflet of the plasma membrane in an ATP-dependent manner [37-45].

Intriguingly, viable, non-apoptotic cancer cells display increased surface PS compared to normal cells [11-14, 19, 23]. Macrophages express receptors for PS and recognize PS that is being exposed on apoptotic cells [34-36]. However, macrophages fail to phagocytose tumor cells due most likely to the high expression of CD47, which inhibits tumor cell phagocytosis [46-48]. Besides this, not much is known about cancer cell surface PS exposure and its biological functions. Understanding the molecular pathways involved in PS exposure in cancer cells, may thus provide novel therapeutic targets to treat cancer. Eventually these studies may facilitate targeted induction of surface PS, especially in low surface PS cancer types, enabling efficient targeting by PS-selective drugs like SapC-DOPS.

In the present study we analyzed human cancer cells from diverse origins, including H1299 (lung cancer), U87ΔEGFR-Luc (glioblastoma), MDA-MB-231(breast cancer), MDA-MB-231-Luc-D3H2LN (metastatic breast cancer), Gli36 (glioblastoma), U373 (astrocytoma) and untransformed human Schwann cells, for surface PS levels and underlying molecular mechanisms controlling PS exposure. We show that cancer cells exhibit varied levels of surface PS, and demonstrate for the first time, the important role for flippase activity in the control of surface PS in cancer cells. We also show that cancer cells differ with respect to intracellular calcium, and that their surface PS exposure is calcium dependent. Furthermore, cancer cell types differ in total cellular PS content, which may in part account for the variations in surface PS.

## RESULTS

### Human cancer cell types differ in the extent of exposed PS on the exoplasmic face of their plasma membranes

**Figure 1:**
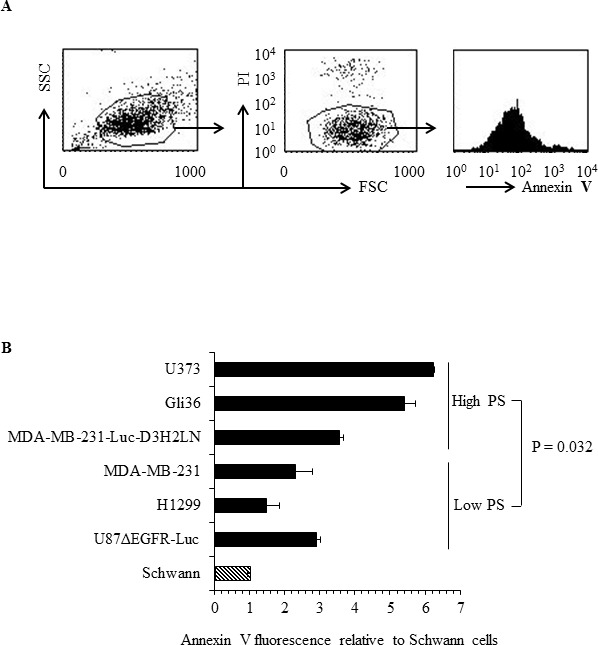
Surface PS exposure on viable human cancer cells **A.** Representative FACS profile of the annexin V FITC binding analyses. Left panel shows forward vs side scatter, middle panel shows gating on live cells by excluding PI positive dead cells. Right panel shows annexin V FITC profile. **B.** Geometrical mean fluorescence signal of annexin V FITC signal from indicated cell types relative to Schwann cells.

To determine the exposure levels of PS on the outer surface of cancer cells, human cancer cell lines and untransformed human Schwann cells were analyzed by flow cytometry for annexin V positivity using FITC-labeled annexin V. Annexin V FITC staining was done in the presence of propidium iodide (PI) to exclude dead cells from analyses (Figure 1A). The indicated PS levels (annexin V FITC fluorescence levels) are thus for PI negative, viable tumor cells (Figure 1A). As shown in Figure 1B, striking differences were observed in the extent of exposed surface PS among different human cancer cell types, with H1299 (lung cancer), U87ΔEGFR-Luc (glioblastoma), MDA-MB-231(breast cancer) exhibiting low and MDA-MB-231-Luc-D3H2LN (metastatic breast cancer), Gli36 (glioblastoma) and U373 (astrocytoma) expressing high surface PS levels. In contrast to cancer cells, untransformed human Schwann cells exhibited the lowest surface PS (Figure 1B). Taken together, flow cytometric analyses for surface PS indicate that cancer cell types differ with respect to surface PS and contain overall elevated PS when compared to normal cells.

### Differential flippase activity regulates surface PS in human cancer cells

**Figure 2:**
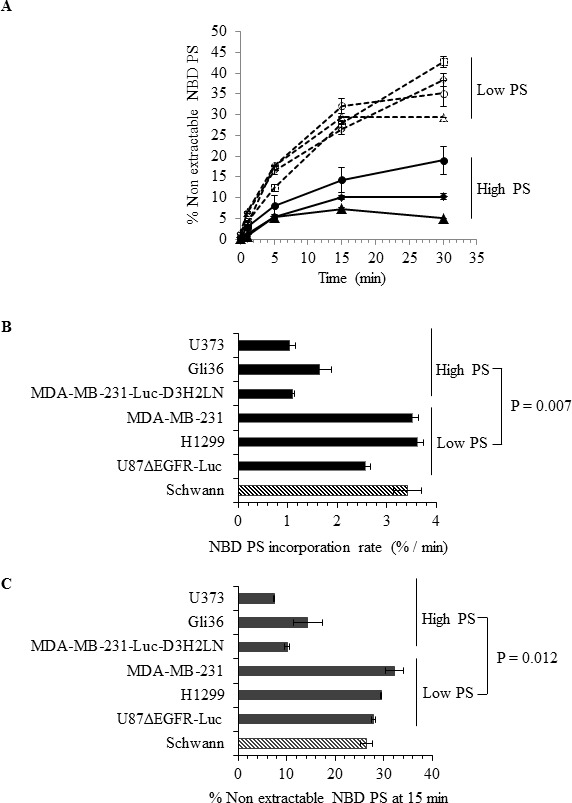
Analyses of flippase activity in different human cancer cell types **A.** Flippase activity assay: Indicated cell types were incubated with NBD-PS for indicated time periods and subjected to BSA extraction and sodium dithionite treatment. % nonextractable NBD-PS (after BSA extraction and sodium dithionite treatment) represents internalized NBD-PS, indicative of flippase activity (Schwann ◊, U87ΔEGFR-Luc □, H1299 Δ, MDA-MB-231 ○, MDA-MB-231-Luc-D3H2LN ◆, Gli36 ▲, U373 ●) **B.** NBD PS incorporation rate. **C.** NBD PS incorporation at 15 minutes time point.

**Figure 3:**
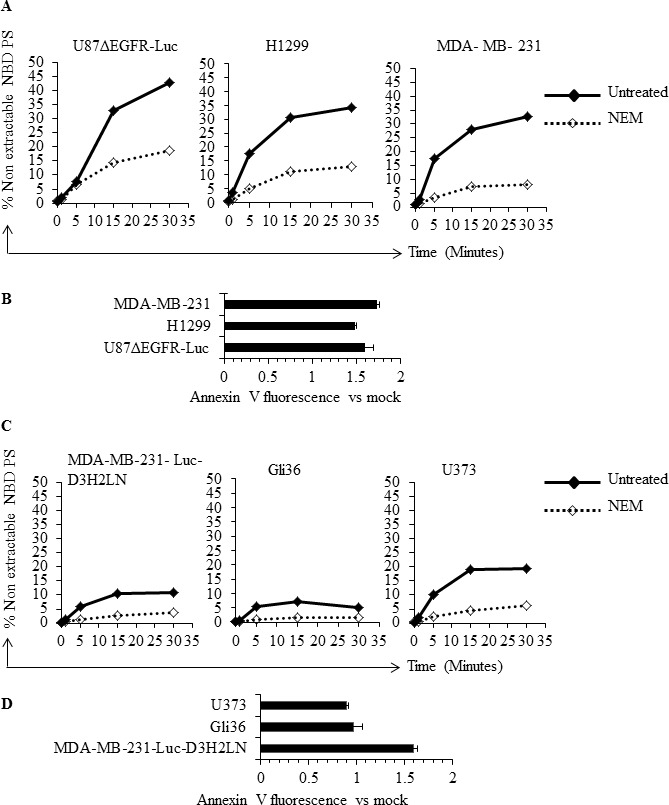
Inhibition of flippase activity by NEM reveals involvement of flippase activity in the regulation of surface PS **A**. Low surface PS cells were either treated with NEM or left untreated, incubated with NBD-PS for indicated time periods and subjected to BSA extraction and sodium dithionite treatment. % nonextractable NBD-PS (after BSA extraction and sodium dithionite treatment) represents internalized NBD-PS, indicative of flippase activity **B**. Flippase activity was inhibited by use of NEM in low surface PS cell lines and surface PS levels were measured by annexin V FITC binding assay, by flow cytometry. The graph shows annexin V FITC fold change compared to mock treated cells. **C**. High surface PS cells were either treated with NEM or left untreated and incubated with NBD-PS for indicated time periods and subjected to BSA extraction and sodium dithionite treatment. % nonextractable NBD-PS (after BSA extraction and sodium dithionite treatment) represents internalized NBD-PS, indicative of flippase activity **D**. Flippase activity was inhibited by use of NEM in the high surface PS cell lines and surface PS levels were measured by annexin V FITC binding assay by flow cytometry. The graph shows annexin V FITC fold change compared to mock treated cells.

The presence of elevated and variable levels of surface PS in non-apoptotic cancer cells is intriguing because normal, non-apoptotic cells do not expose PS on their surface; this suggests a probable dysregulation of the molecular machinery involved in the maintenance of plasma membrane PS asymmetry. Because flippases are critical regulators of PS localization to the inner leaflet of the plasma membrane [37-43], we examined flippase activity in cancer cell lines with either high or low surface PS and in untransformed Schwann cells. Flippase activity was measured as the rate of translocation of the fluorescent PS analogue NBD-PS (1-palmitoyl-2-{6-[(7-nitro-2-1,3-benzoxadiazol-4-yl)amino]hexanoyl}-*sn*-glycero-3-phosphoserine (ammonium salt), from the outer to the inner leaflet of the plasma membrane. Cells were incubated with NBD-PS for the indicated times, the non-translocated NBD-PS was extracted from the outer leaflet by BSA and reduced by sodium dithionite treatment, and fluorescence was measured by flow cytometry and expressed as percentage of nonextractable NBD-PS fluorescence (Figure 2A). Under these conditions, the measured fluorescence corresponds to the NBD-PS translocated to the inner leaflet and thereby reflects the flippase activity. An inverse correlation was observed between flippase activity and surface PS, with overall high flippase activity in non-transformed Schwann cells and tumor cell types with low surface PS (U87ΔEGFR-Luc, H1299, MDA-MB-231), and low flippase activity in cancer cell lines with high surface PS (Gli36, U373) (Figure 2A). Both the initial rate of incorporation and the amount of NBD-PS internalized after 15 min were higher in low surface PS cells (Figures 2B and 2C). Inhibition of cellular flippase activity by N-ethylmaleimide (NEM) [37, 38, 44] led to elevation of surface PS in low surface PS cancer cell lines, suggesting that in these cells high flippase activity indeed plays a role in maintaining low surface PS (Figure 3B). In contrast, NEM had little or no effect on high surface PS Gli36 and U373 cells, which exhibit low baseline flippase activity (Figure 3D). To confirm whether NEM -dependent elevation of surface PS in low surface PS cells is a true reflection of inhibition of flippase activity, flippase assays were performed as described above. As shown in Figure 3A, in low surface PS cells NEM treatment led to marked reduction in flippase activity compared to untreated cells. In high surface PS cells (Gli36, U373), basal, low flippase activity was also inhibited by NEM treatment (Figure 3C), but surface PS levels did not change except in the cell line with intermediate PS exposure levels (MDA-MB-231-Luc-D3H2LN), where it increased to an extent similar to that of cells with low basal surface PS levels (Figure 3D). These results indicate that in cultured cancer cells high flippase activity helps maintain low PS exposure, whereas low flippase activity predominates in cells displaying moderate to high surface PS levels. Thus, our data implies that surface PS expression in viable cancer cells is at least in part a reflection of basal flippase activity.

### Total cellular PS contributes to surface exposure of PS

**Figure 4:**
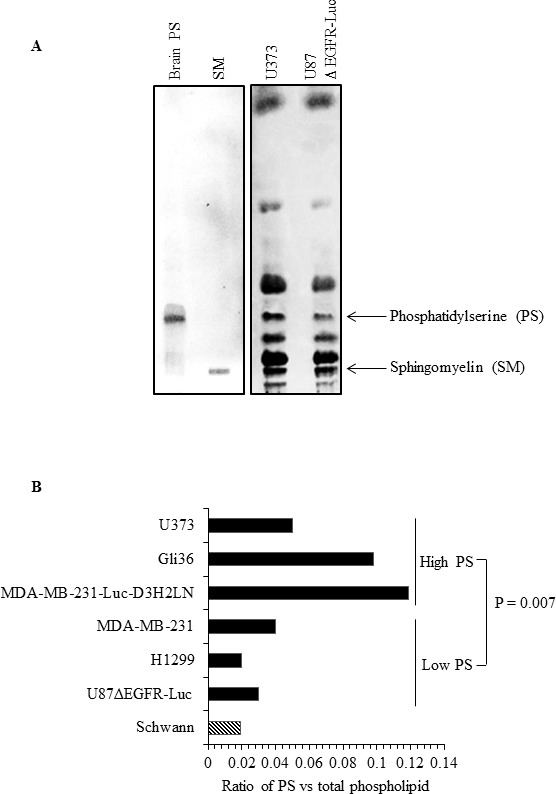
Human cancer cells differ in total cellular PS content **A**. TLC profile of phospholipids from U87ΔEGFR-Luc and U373 cell lines, with purified brain PS and SM run as molecular standards. **B**. Total PS estimated in the indicated cell lines by phosphorus assay from scraped PS bands obtained from TLC, expressed as the ratio of phosphorous obtained from scraped PS band and phosphorous from total phospholipids.

**Figure 5:**
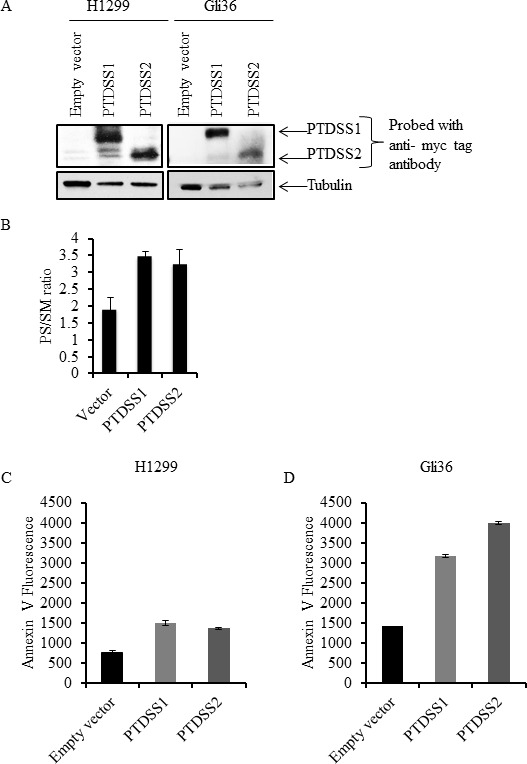
Overexpression of PS synthases PTDSS1 and PTDSS2 in human cancer cell lines increases surface PS **A**. Whole cell lysates from H1299 and Gli36 cells transfected with pIRESneo3/empty vector, pIRESneo3/myc-PTDSS1 or pIRESneo3/myc-PTDSS2 vectors were separated by SDS-PAGE and proteins were transferred on to nitrocellulose membrane. Expression of PTDSS1 and PTDSS2 was detected using anti-myc tag antibody. **B**. Lipids from H1299 cells transfected with indicated vectors and selected with G418, were separated by TLC and variations in PS are shown as the ratio of PS to SM. Transfected cells were stained with annexin V FITC to measure surface PS exposure. Annexin V FITC fluorescence was acquired by flow cytometry and its geometrical mean is shown for H1299 **C**. and Gli36 **D**. cells.

Since the amount of external PS in cancer cells may depend on the total amount of cellular PS, we determined total PS in different cancer cell types and in normal Schwann cells. Lipids were extracted and subjected to separation by TLC, using purified PS and sphingomyelin (SM) as molecular standards (Figure 4A). As shown in the sample TLC profile (Figure 4A), total PS was higher in U373 cells (high surface PS) compared to low surface PS U87ΔEGFR-Luc cells. Interestingly, in accordance with previous studies [49], in addition to differences in cellular PS we observed alterations in the abundance of other phospholipids (Figure 4A; lipid content normalized to protein content). Phospholipids from low surface PS (U87ΔEGFR-Luc, H1299, MDA-MB-231), or high surface PS (MDA-MB-231-Luc-D3H2LN, Gli36, U373) cancer cell lines, and from normal Schwann cells, were separated by TLC. Total PS was then estimated by extraction of phosphorus from scraped PS bands and expressed as the fraction of total phospholipid phosphorus. This quantification showed higher overall total PS levels in cancer cells compared to normal Schwann cells, and showed also that high surface PS cancer cell lines had more total PS compared to low surface PS cell types (Figure 4B). Estimation of PS by calculating the ratio of PS to sphingomyelin (SM) from band intensities from TLC plates yielded similar results, namely higher cellular PS content in high surface PS U373 cells compared to low surface PS U87ΔEGFR-Luc cells (Supplementary Figure 1). To directly address whether increased cellular PS affects surface PS exposure, we overexpressed myc-tagged PS synthases PTDSS1 and PTDSS2 in H1299 and Gli36 cells, because these are the two enzymes known so far in PS synthesis [50]. Expression of PTDSS1 and PTDSS2 was verified by using anti-myc tagged antibody (Figure 5A). Overexpression or gain-of-function mutations in PS synthases have been shown to increase PS synthesis [49, 51]. Accordingly, TLC analyses of lipids from H1299 cells revealed increased cellular PS levels in cells transfected with PTDSS1 and PTDSS2 compared to empty vector transfected cells (Figure 5B). Interestingly, in both PTDSS1- and PTDSS2-overexpressing H1299 and Gli36 cells, surface PS exposure was elevated compared to control cells transfected with an empty vector (Figures 5C and 5D). This result indicates that increased expression of PS synthesizing enzymes leads to increased cellular PS levels, suggesting that total cellular PS influences surface PS exposure.

### Intracellular calcium levels regulate surface PS exposure in human cancer cells

**Figure 6:**
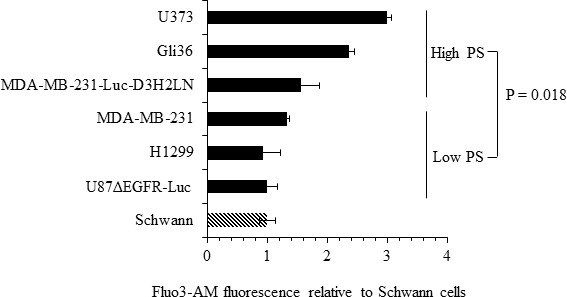
Intracellular calcium levels in cancer cells Cells were loaded with the calcium binding dye Fluo-3 AM and fluorescence was measured by flow cytometry. Shown are the fold changes in Fluo-3 AM fluorescence compared to untransformed Schwann cells.

**Figure 7:**
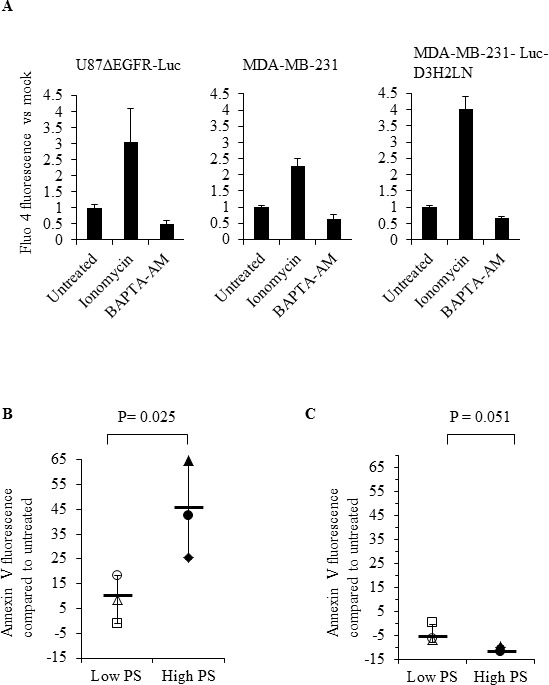
Modulation of intracellular calcium by ionomycin or BAPTA-AM affects surface PS in human cancer cells **A**. Cells were treated with DMSO, ionomycin or BAPTA-AM, incubated with the calcium binding dye Fluo-4 Direct and fluorescence was measured using a microplate reader. Bars show relative changes in Fluo-4 Direct fluorescence signal for each cell line normalized to DMSO controls. **B**. Low and high surface PS cells were treated with DMSO or ionomycin and surface PS levels were assayed by staining with annexin V FITC, followed by flow cytometry. The axis indicates shift in the geometrical mean of annexin V FITC fluorescence in ionomycin treated vs untreated cells (U87ΔEGFR-Luc □, H1299 Δ, MDA-MB-231 ○, MDA-MB-231-Luc-D3H2LN ◆, Gli36 ▲, U373 ●, Mean—). (C) Low and and high surface PS cells were treated with DMSO or BAPTA-AM, followed by incubation with annexin V FITC and analyzed by flow cytometry. The axis indicates shift in the geometrical mean of annexin V FITC fluorescence in BAPTA-AM treated vs untreated cells (U87ΔEGFR-Luc □, H1299 Δ, MDA-MB-231 ○, MDA-MB-231-Luc-D3H2LN ◆, Gli36 ▲, U373 ●, Mean—).

The role of intracellular calcium in cancer has been well documented [52, 53]. Calcium inhibits flippase activity, and elevated calcium causes PS externalization in human erythrocytes [39, 45]. Therefore, we assessed if calcium levels correlate with surface PS expression in cancer cells, by measuring intracellular calcium levels in cancer and normal cells using the calcium sensitive fluorescent dye Fluo3-AM (Figure 6). We observed steady state intracellular calcium levels to be higher in high (Gli36, U373, MDA-MB-231-Luc-D3H2LN) compared to low (U87ΔEGFR-Luc, MDA-MB-231, H1299) surface PS cancer cells as well as normal Schwann cells (Figure 6). Next, we determined if intracellular calcium levels in different cancer cell types indeed play a role in surface PS maintenance. To this end we treated low and high surface PS cancer cells with ionomycin to elevate intracellular calcium levels and confirmed increase in intracellular calcium by using calcium sensitive fluorescent dye Fluo-4 Direct (Figure 7A). Changes in cell surface PS upon ionomycin treatment were monitored by annexin V FITC staining and flow cytometry. As shown in Figure 7B, ionomycin treatment increased, albeit modestly, surface PS levels in both high and low surface PS cell lines. This suggests that intracellular calcium levels indeed regulate surface PS expression, although to varying extents (Figure 7B). To test if steady state intracellular calcium affects surface PS exposure, cells were treated with BAPTA-AM to chelate intracellular calcium (Figure 7A). As shown in Figure 7C, depletion of intracellular calcium led to a slight decline in surface PS levels. These results indicate that fluctuations in intracellular calcium may affect surface PS exposure in human cancer cells.

## DISCUSSION

In this study we examined potential mechanisms for PS exposure in different cancer cell types and found that low flippase activity, increased calcium, and increased total PS contribute to increased surface PS exposure. We found that cancer cell lines from diverse origins differ in the extent of surface PS exposure (Figure 1B), although a correlation between tumor origin and surface PS could not be established by our study. Because flippases are critical controllers of membrane PS asymmetry, we first analyzed if the activity of these enzymes differs in cancer cells with either low or high surface PS [37-43]. We observed large differences in flippase activity among different cell types and found that low surface PS is in general associated with high flippase activity, while high surface PS cancer cell types contain low flippase activity (Figures 2A-2C). The correlation between surface PS exposure and flippase activity was further evident upon inhibition of flippase activity by NEM (Figures 3B and 3D), which led to surface PS elevation in low surface PS cancer cells, suggesting that in these cells high flippase activity continuously transfers PS from the outer to the inner leaflet of the plasma membrane (Figure 3B). In contrast, in cell lines with the highest surface PS levels PS exposure remained unaltered by NEM, suggesting little endogenous flippase activity in these cells (Figure 3D). Both these results indicate the critical involvement of flippase activity in determining low vs high surface PS exposure in cancer cells. As the effects observed in MDA-MB-231-D3H2LN, a metastatic breast cancer cell line showing moderately high surface PS levels, suggest, it is likely that in addition to low or high flippase activity, other membrane PS-translocating mechanisms, such as phospholipid scramblases and floppases, also play a role in determining PS exposure in viable cancer cells. These results strongly suggest that a primary cause for elevated PS exposure in cancer cells is the differential activity of flippases, which may in turn depend on the differential expression of these enzymes.

Another possible contributing factor for elevated PS exposure in cancer cells is the presence of high total cellular PS. Our analyses indicate that in general total cellular PS levels were higher in cancer cells compared to untransformed cells (Figures 4A and 4B). Among tested cancer cells, total PS levels were higher in high surface PS cell lines, suggestive of a correlation between cellular and surface PS levels (Figures 4A and 4B). In addition to PS, we observed variations in the abundance of other phospholipids between high and low surface PS cancer cells, which may be indicative of the interdependency of phospholipid synthesis [49, 50]. On the other hand, overexpression of PS synthases PTDSS1 and PTDSS2 in H1299 and Gli36 cells, and the subsequent elevation of surface PS in these cells, suggests cellular PS synthesis status as one of the regulatory factors in the control of surface PS (Figures 5A-5D). In this regard, future studies should focus on addressing the expression status and activity of PS synthases PTDSS1 and PTDSS2 in different tumors, which might reveal important information in terms of mis-regulation of these enzymes in cancer.

Because calcium is a known regulator of flippase activity [37, 39, 45], differences in cellular calcium may be pivotal in the regulation of surface PS. Accordingly, we found varied levels of calcium in different cancer cell lines and a positive correlation between intracellular calcium and PS exposure was observed (Figure 6). Induced alterations in steady state intracellular calcium by ionomycin or by chelation further provided evidence for surface PS regulation by calcium (Figures 7B and 7C). Elevation of intracellular calcium by ionomycin led to an increase in surface PS in both low and high surface PS cancer cell types, with the latter responding more strongly. Conversely, calcium chelation led to lower surface PS in both high and low surface PS cancer cells. These results suggest the importance of calcium in the regulation of surface PS in cancer cells.

**Figure 8:**
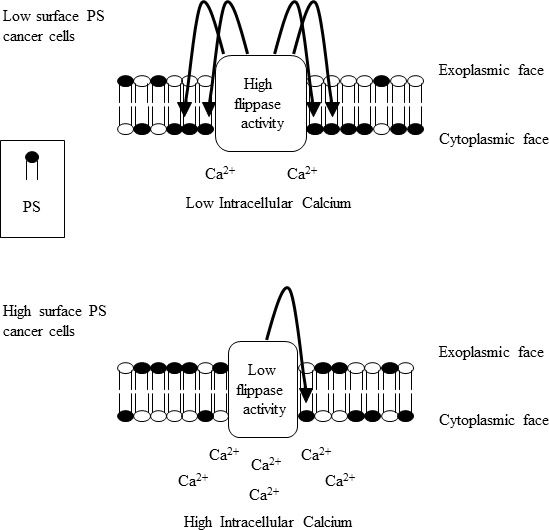
Model summarizing the alterations in cytoplasmic calcium and flippase activity in surface PS low and high cancer cells Surface PS low cancer cells contain low cytoplasmic calcium and high flippase activity, culminating in lower surface PS exposure (upper panel). Surface PS high cancer cells contain high cytoplasmic calcium and reduced flippase activity resulting in higher surface PS exposure (lower panel).

In summary, our study interrogates the mechanisms of abnormal PS exposure in viable cancer cells and reveals an inverse correlation between flippase activity and constitutive PS externalization. Steady-state calcium levels, possibly by inhibiting flippase activity, also affect PS exposure and constitute another influencing factor of altered PS exposure in cancer cells (Figure 8). Insights into PS exposure mechanisms in cancer cells might facilitate tumor cell specific induction of surface PS, and thus enhance the effectiveness of PS targeting drugs such as SapC-DOPS and anti-PS antibodies [15, 16, 18, 19, 22, 23, 54]. Future studies addressing the molecular basis for alterations in the activity/expression of flippases and other phospholipid-translocating enzymes, and the nature of variations in intracellular calcium levels in different types of cancer, will shed more light on cancer's basic biology and open up new therapeutic opportunities to treat this disease.

## MATERIALS AND METHODS

### Cell lines and cell culture

Human cancer cell lines MDA-MB-231, H1299, Gli36 and U373 were obtained from ATCC (Manassas, VA, USA). MDA-MB-231-luc-D3H2LN cells were obtained from Caliper Life Sciences, Mountain View, CA. U87ΔEGFR-Luc cells were obtained from Dr. Balveen Kaur, Ohio State University, Columbus, Ohio. Human Schwann cells were obtained from ScienCell (Carlsbad, CA, USA). U87ΔEGFR-Luc, Gli36 and U373 were cultured in DMEM medium (Fisher Scientific). H1299 was cultured in RPMI medium (Fisher Scientific). MDA-MB-231 non metastatic and MDA-MB-231- Luc-D3H2LN metastatic cell lines were cultured in AMEM medium (Invitrogen). The above cell lines were cultured in their respective media supplemented with 10% FBS and 1% Penicillin / Streptomycin. Normal human Schwann cells were cultured in Schwann cell medium (ScienCell, Carlsbad, CA, USA) supplemented with the provided growth factor supplement, FBS and antibiotics. All cells were cultured in a 5% CO_2_ incubator at 37°C. Cells were routinely tested for mycoplasma contamination. No cross-contamination was observed in the used cell lines, as evidenced by the cellular morphology and growth parameters. No authentication of the cell lines was done by the authors.

### Flow cytometric analyses of annexin V binding

Cells at 70% confluency were trypsinized, resuspended in complete medium, spun down and washed once with annexin V binding buffer. Cells (1 × 10^5^) were incubated with 5 μl annexin V FITC (Invitrogen) and 2 μg/ml propidium iodide (PI) in a final volume of 100 μl at room temperature in dark for 25 minutes. AnnexinV FITC binding was measured by flow cytometry after adding 500 μl of annexin V binding buffer, using BD FACS calibur or BD Fortessa. Data was analyzed by Cell quest programme or BD FACS Diva. For analyzing the annexin V FITC signal from living cells, PI positive dead cells were gated out and annexin V FITC signal was obtained from PI negative forward scattered cells.

### Flippase assay

Cells, untreated or treated with NEM 500 μM (Sigma) for 10 minutes, were washed once with PBS and once with flippase assay buffer (20 mM Hepes pH 7.6, 10 mM glucose, 45 mM NaCl, 100 mM KCl, 0.2 mM MgCl_2_). Cells (3 × 10^6^) were resuspended in 3ml of flippase assay buffer and NBD-PS (Avanti Polar Lipids) phospholipids added to 3 μM final concentration. Cells with NBD-PS were divided into 500 μl aliquots and incubated for 0, 1, 5, 15 and 30 minutes. After each incubation time, half of the cell suspension was separated for non-extracted sample and kept on ice. The remaining half was spun down to remove non inserted NBD-PS and subjected to BSA extraction of NBD-PS from the outer leaflet by adding 3% fatty acid free BSA (MP biomedicals) in flippase assay buffer. After incubation on ice for 20 minutes, freshly prepared sodium dithionite (Sigma) was added to a final concentration of 10 mM and incubated a further 10 minutes, to reduce the NBD lipids in the outer cell surface. The cells were spun down and resuspended in flippase assay buffer containing 0.25% BSA and 2 μg/ml PI. NBD-PS signal from unextracted and extracted samples was measured by flow cytometry from living cells after exclusion of PI-positive dead cells, using BD FACS Calibur or BD Fortessa. Non-extractable NBD-PS in the BSA and sodium dithionite treated sample was presented as the percentage of total amount in the control unextracted sample.

### Thin layer chromatography (TLC) and quantification of phosphatidylserine

Total cellular lipids from indicated cells were extracted by chloroform/methanol extraction. TLC was performed as previously described [55]. Equal amounts of lipids were loaded onto a TLC plate based on protein quantification from individual cell lines and lipids were separated by TLC. Brain PS (Avanti Polar Lipids) and SM (Matreya LLC) were run as molecular standards. Bands corresponding to brain PS were scraped and subjected to phosphorus extraction by acidic digestion. The liberated phosphorus was estimated by allowing a complex formation with ammonium molybdate (Sigma) and malachite green (Sigma) and by measuring the absorption at 660 nm [56]. Phosphorus was quantified using a standard curve obtained from phosphorus liberated from known concentrations of brain PS run on TLC plate. Cellular PS was expressed as the ratio of phosphorus obtained from PS and phosphorus obtained from total phospholipids. Additionally, PS was estimated by acquiring TLC band intensities of PS and sphingomyelin (SM), using Image Studio Lite software; variations in PS are shown as a ratio of PS to SM bands.

### Transfection of cells with PS synthase 1 (PTDSS1) and PS synthase 2 (PTDSS2)

H1299 and Gli36 cells grown to 80% confluence were transfected individually with 3 μg DNA of either pIRESneo3/empty vector, pIRESneo3/myc-PTDSS1 or pIRESneo3/myc-PTDSS2 vectors (kind gifts from Dr. Shin-ya Morita, Japan), in 6 cm culture dishes, using lipofectamine 3000 (Invitrogen), according to the manufacturer's instructions. Transfected cells were selected with G418 (Life technologies), using a concentration of 1mg/ml for H1299 cells and 500 μg/ml for Gli36 cells.

### Western blotting

Whole cell extracts from H1299 and Gli36 cells transfected with pIRESneo3/empty vector, pIRESneo3/myc-PTDSS1 or pIRESneo3/myc-PTDSS2 vectors, were prepared by using RIPA buffer (Sigma) and equal amounts of proteins (25 μg each) were separated by SDS-PAGE followed by western blotting. Expression of Myc-tagged PTDSS1 and PTDSS2 proteins was detected by using an anti-myc tag antibody (Cell Signaling). Anti- tubulin antibody (Novus biologicals) was used to detect tubulin expression.

### Measurement of intracellular calcium

Intracellular calcium was measured by using the calcium binding dyes Fluo-3 AM and Fluo-4 Direct (Invitrogen). Essentially, cells (1 × 10^5^) were loaded with 5 μM final concentration of Fluo-3 AM and incubated for 30 min at 37°C. Cells were washed twice with culture medium, resuspended in medium and incubated for a further 30 minutes at 37°C. Cells were washed twice, 2 μg/ml PI was added and Fluo-3 AM signal was measured by flow cytometry after exclusion of PI positive dead cells, using BD FACS calibur. Data was analyzed using CellQuest software. For measurement of intracellular calcium levels by Fluo-4 Direct, cells (0.3 × 10^5^) in 100 μl medium per well were plated in black 96 well plates. The next day, cells were loaded with Fluo-4 Direct reagent to a final concentration of 1X and incubated for 30 minutes in a CO_2_ incubator. Fluorescence was measured using a plate reader with excitation at 494 nm and emission at 516 nm.

### Calcium modulation by ionomycin, BAPTA-AM treatment and annexin V binding measurement

Cells were treated with 5 μM ionomycin (Invitrogen), 10 μM BAPTA-AM (Tocris) or DMSO for 45 minutes, washed once with PBS and once with annexin V binding buffer. Cells (1 × 10^5^) were incubated with 5 μl annexin V FITC and 2 μg/ml PI in a final volume of 100μl at room temperature in the dark for 25 minutes. Annexin V FITC binding was measured by flow cytometry after adding 500 μl of annexin V binding buffer, using BD FACS calibur. Data was analyzed with CellQuest. PI-positive dead cells were gated out and annexin V signal was obtained from PI negative forward scattered cells.

## SUPPLEMENTARY MATERIAL FIGURE



## Abbreviations

PS: phosphatidylserine
SM: Sphingomyelin
NBD-PS: 1-palmitoyl-2-{6-[(7-nitro-2-1,3-benzoxadiazol-4-yl)amino]hexanoyl}-sn-glycero-3-phosphoserine (ammonium salt)
SapC: saposin C
DOPS: dioleoylphosphatidylserine
NEM: N-ethylmaleimide
PTDSS: phosphatidylserine synthase
