# Supplementary material for: Variation in human cancer cell external phosphatidylserine is regulated by flippase activity and intracellular calcium
Source: Oncotarget. 2015 Oct 9;6(33):34375–88. doi: 10.18632/oncotarget.6045 (PMC4741459; doi:10.18632/oncotarget.6045)
Supplement: Supplementary file 1 [file oncotarget-06-34375-s001.pdf]

Variation in human cancer cell external phosphatidylserine is regulated by flippase activity and intracellular calcium

Supplementary Material

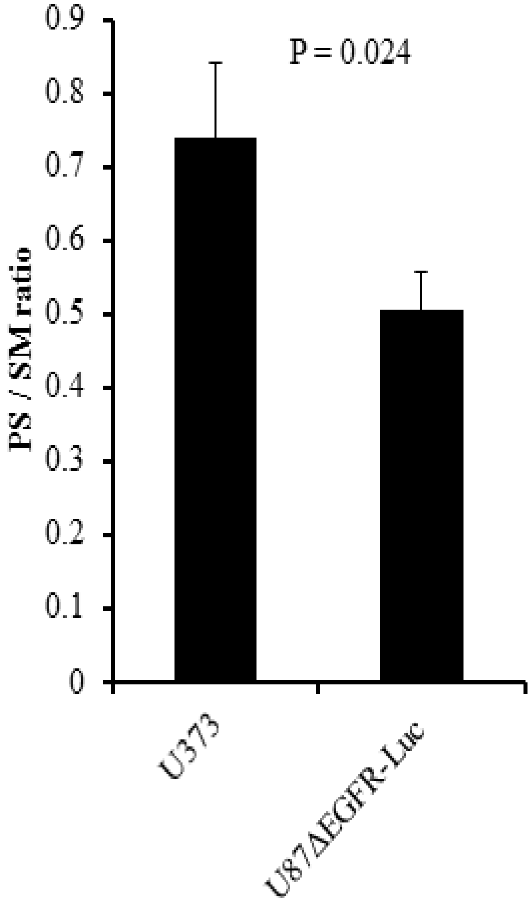

Supplementary Figure 1. Lipids from indicated cell lines were separated by TLC and total PS is presented as a ratio of PS to SM.
